# Single‐nuclei RNA sequencing reveals heterogeneity within developing GnRH3 neurons in zebrafish

**DOI:** 10.1111/jne.70230

**Published:** 2026-07-07

**Authors:** Yalong Sun, Matan Golan, Yonathan Zohar, Xiaoxuan Fan, Nilli Zmora

**Affiliations:** ^1^ Institute of Marine & Environmental Technology, Department of Marine Biotechnology University of Maryland Baltimore County Baltimore Maryland USA; ^2^ Department of Animal Sciences, The Robert H. Smith Faculty of Agriculture, Food, and Environment The Hebrew University of Jerusalem Rehovot Israel; ^3^ Department of Microbiology and Immunology University of Maryland Baltimore Baltimore Maryland USA

**Keywords:** gonadotropin‐releasing hormone neurons, heterogeneity, migration, single‐nuclei RNA sequencing

## Abstract

Gonadotropin‐releasing hormone (GnRH) is a central regulator of vertebrate reproduction regulating pituitary gonadotropins. In zebrafish, GnRH3 serves as the hypophysiotropic isoform. Its neurons develop by migrating from the nasal placode to the hypothalamus, forming several distinct subpopulations along the migratory path. However, the molecular heterogeneity underlying this migration remains incompletely understood. To delineate the molecular and spatial heterogeneity, we employed single‐nuclei RNA sequencing of nuclei enriched based on *gnrh3* promoter‐driven EGFP fluorescence from 7 dpf *Tg(gnrh3:EGFP)* zebrafish. This was followed by GnRH3 neurons clustering, differentially expressed genes identification, trajectory inference as a hypothesis‐generating framework, and functional analyses. Marker genes were selected and used for validation and to determine the distribution of each subcluster by in situ hybridization (ISH) and immunohistochemistry (IHC) in larval and adult zebrafish. Three subpopulations were identified featuring highly expressed marker genes: Gnrh3^cyp1a^, Gnrh3^nrg1^, and Gnrh3^npffl/prl2^. Gnrh3^npffl/prl2^ was further clustered into two subpopulations, Gnrh3^npffl/prl2^ and Gnrh3^npffl^, which localized in the olfactory bulbs/terminal nerve and telencephalon, whereas Gnrh3^cyp1a^ and Gnrh3^nrg1^ were detected in distinct cells within the olfactory epithelium. Trajectory inference and gene ontology enrichment analyses were consistent with a putative transcriptomic continuum from Gnrh3^cyp1a^ to Gnrh3^npffl/prl2^ states, with the latter subpopulation lacking canonical migratory gene signatures. Protein–protein interaction network analysis revealed interaction relationships among differentially expressed genes, with a GnRH3‐associated interaction module further supporting the selection of the above marker genes. Our results support the notion of at least three distinct GnRH3 subpopulations with distinct transcriptomes and characteristics, providing a broader basis for deciphering the diverse roles of GnRH3 in migration and reproduction.

## 1 INTRODUCTION

The hypothalamus‐pituitary‐gonadal axis controls reproduction in all vertebrates, with gonadotropin releasing hormone (GnRH) being the upstream regulator. GnRH regulates the release of luteinizing hormone (LH) and follicle‐stimulating hormone (FSH) from the pituitary, which in turn regulate the function of the gonads.^1^, ^2^

The number of GnRH isoforms ranges from one in rodents to three in many teleost fishes. The preoptic area (POA) GnRH1 (=LHRH) isoform is essential for reproduction, through innervating the pituitary.^1^ Mutations in GnRH1 or its receptor genes lead to infertility in mammals.^3^, ^4^ In fish that possess three GnRH isoforms, GnRH1 neurons populate the preoptic area (POA), GnRH2 neurons the midbrain tegmentum, and GnRH3 neurons the telencephalon (TEL) and olfactory bulbs/terminal nerve (OB/TN). GnRH3, a teleost‐specific isoform, exerts neuro‐modulatory effects throughout the brain.^5^, ^6^, ^7^ In fish with two GnRH isoforms, such as zebrafish, GnRH3 is the preoptic hypophysiotropic form,^8^, ^9^ considered to be analogous to GnRH1.^10^ In teleosts, hypophysiotropic regulation of pituitary gonadotropin secretion is increasingly viewed as a coordinated network process. In addition to GnRH peptides, other neuropeptides (including cholecystokinin) have been proposed to contribute to differential regulation of LH‐ and FSH^11^; thus, functional assignments and nomenclature continue to evolve.

Similar to GnRH1 neurons in other vertebrates, GnRH3 neurons in zebrafish undergo a long developmental migration process, originating from the nasal placode at approximately 24 h post fertilization (hpf),^12^ and migrate to the nasal‐forebrain junction (NFJ) where they form inter‐hemispheric circuit before entering the brain.^12^ During their migration, GnRH neurons traverse diverse extracellular environments associated with changing modes. Until recently, the main paradigm was that the newborn GnRH neurons are uniform, migrating in a continuous flow along the forebrain, stopping as dictated by surrounding external cues.

To reveal the different subpopulations and spatiotemporally and molecularly characterize them, we employed a single‐nuclei RNA sequencing (snRNA‐Seq) of enriched GnRH3 neuronal nuclei of 7 dpf *Tg(gnrh3:EGFP)*. Transcriptomic relationships among GnRH3 neuronal nuclei and protein–protein interaction (PPI) networks of differentially expressed genes (DEGs) were examined. Four candidate gene markers (*neuropeptide FF‐amide peptide precursor like* (*npffl*), *prolactin 2* (*prl2*), *neuregulin 1* (*nrg1*), and *cytochrome P450*, *family 1*, *subfamily A* (*cyp1a*)), representing distinct GnRH3 subclusters, were selected and their co‐expression in GnRH3 neurons was verified using *in situ* hybridization (ISH)/immunohistochemistry (IHC) in 7 dpf larvae and adult zebrafish. Our findings support the presence of at least three molecularly distinct GnRH3‐associated subpopulations/states with distinct transcriptomes in zebrafish.

## 2 RESULTS

### 2.1 Identification of subclusters of nuclei co‐expressing *gnrh3* and marker genes

**FIGURE 1:**
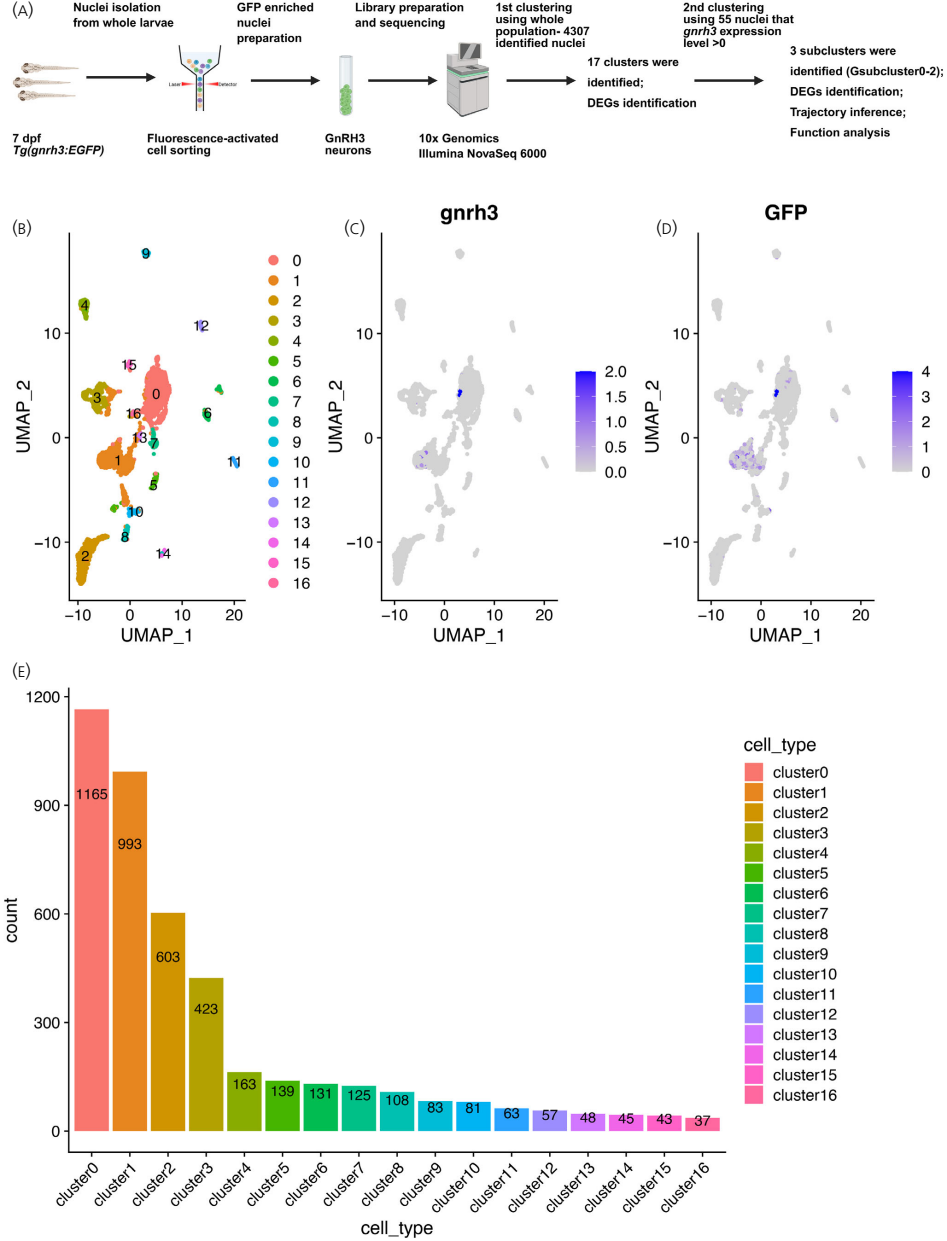
Single‐nuclei RNA sequencing of 4307 EGFP‐enriched nuclei isolated from 7 dpf *Tg(gnrh3:EGFP)* zebrafish. (A) Schematic diagram of the snRNAseq workflow (created with BioRender.com). (B) First round single‐cell uniform manifold approximation and projection (UMAP) plot shows 17 clusters formed from the 4307 EGFP‐enriched nuclei. UMAPs display relative expression and distribution of *gnrh3* (C) and GFP (D). (E) The number of nuclei sequenced in each cluster.

In order to identify the different subpopulations of GnRH3 neurons during migration, snRNA‐Seq was applied on 4307 enriched EGFP‐labeled nuclei isolated from *Tg(gnrh3:EGFP)* 7 dpf larvae (Figure 1A). These 4307 nuclei represent the post‐library, post‐QC dataset derived from the EGFP‐enriched FACS fraction, not the total number of sorted events. Seventeen clusters were identified, of which cluster 0 and 1 expressed both *gnrh3* and *GFP* significantly higher than the other clusters (Figure 1B–D). Cluster 0, 1, 2, and 3 comprised the majority of the nuclei with 1165, 993, 603, and 423 nuclei, respectively (Figure 1E). Top 30 differentiated marker genes in clusters 0–3 and the relative differential expression/distribution of top 10 of them in these four dominant clusters are listed in Tables S1–S4 and Figures S1–S4 (Supporting Information), respectively.

**FIGURE 2:**
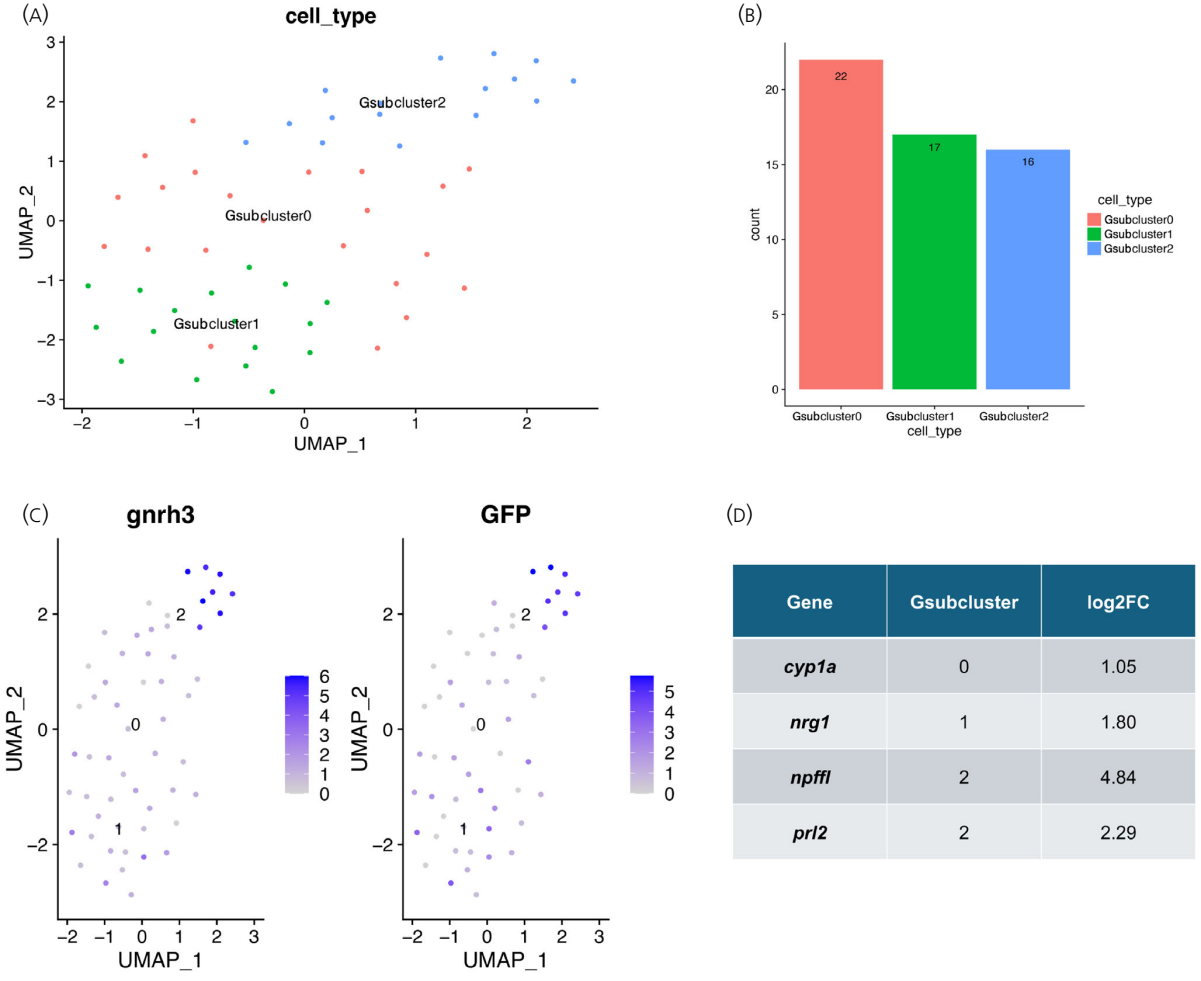
Second round analysis was performed on 55 nuclei with detectable nuclear gnrh3 transcripts. (A) Single‐cell uniform manifold approximation and projection (UMAP) plot formed 3 subclusters. (B) The number of nuclei sequenced in each subcluster. (C) UMAPs display relative expression and distribution of *gnrh3* (left) and *GFP* (right) in all 55 nuclei. (D) Selected marker genes and their relative expression in the three identified GnRH3 subclusters for downstream validation.

Reclustering was conducted using the Seurat object displaying *gnrh3* expression level >0 (Figure 1A). We identified three subclusters, GnRH3‐subcluster0‐2 (Gsubcluster0‐2), harboring 22, 17, and 16 nuclei, respectively (Figure 2A,B), expressing *gnrh3* and *GFP*. Gsubcluster2 exhibited higher *gnrh3* and *GFP* mRNA prevalence/nuclei (Figure 2C). The 4307 nuclei analyzed here represent the post‐library, post‐quality‐control dataset derived from the approximately 10,000 nuclei taken from the EGFP‐enriched FACS fraction for cDNA library preparation, rather than the total number of FACS‐sorted events. Within this dataset, 55 nuclei showed detectable nuclear *gnrh3* transcripts and were retained as a gnrh3‐detectable subset for exploratory subclustering. The number of GnRH3 neurons in zebrafish at 7 dpf is approximately 20 per larva, representing about 0.02% of the total neuronal population (~100,000 neurons) in the larval zebrafish brain.^13^, ^14^, ^15^ This proportion would be even smaller when considering the total body cell number. Thus, the 55 (=1.28% of 4307) identified GnRH3 nuclei represent a ~64‐fold enrichment. However, losses are expected during whole‐larvae nuclei isolation, filtration, FACS, 10X loading, library preparation, and post‐sequencing quality control. Therefore, the recovery of 55 nuclei with detectable nuclear gnrh3 transcripts should be interpreted as a molecular subset within an EGFP‐enriched dataset rather than as the total number of GnRH3‐lineage nuclei present in the sorted material.

The top 30 differentially expressed genes (DEGs) are listed for each Gsubcluster (Tables S5–S7). The functional categorization of 22 genes associated with GnRH neurons revealed substantial overlap among five key biological categories: GnRH neuron development (Dev), neuron migration (Mig), secretion and function (Reg), axon guidance and synaptic connectivity (Guid), and Kallmann syndrome or related CHH (KS_CHH).^16^, ^17^ The largest intersection comprised six genes (*il17rd*, *dusp6*, *spry4*, *hesx1*, *lhx4*, *sox2*) that were concurrently assigned only to both Dev and KS_CHH (Figure S5). *Rnf216*, *stub1*, *dmxl2*, *lepr*, and *pcsk1* were uniquely classified under both Reg and KS_CHH, while *notch*, *chd7*, and *wdr11* were shared exclusively among Dev, Mig, and KS_CHH functional categories (Figure S5). Another distinct subset included *polr3a*, *polr3b*, *pnpla6* that were exclusively categorized under KS_CHH, suggesting limited or syndrome‐specific functionality. Single‐gene intersections were also observed: *flrt3* was assigned to Dev, Guid, and KS_CHH; *sema7a* to Mig, Guid, and KS_CHH; *nrg1* to Reg and Guid; *mki67* solely to Dev; and *fgf13a* solely to Mig (Figure S5). These genes show variable expression levels and distribution across the different Gsubclusters. Gsubcluster0 and Gsubcluster1 exhibited high *notch* and *nrg1* mRNA prevalence (Figure S6A,C). Contrastingly, the expression of *fgf13a* is relatively high in a portion of the cells in Gsubcluster2, while *il17rd*, *dusp6*, *spry4*, *flrt3*, *sema7a*, *hesx1*, *chd7*, *wdr11*, *rnf216*, *polr3a*, *polr3b*, *pnpla6*, *stub1*, *dmxl2*, *lepr*, *lhx4*, *pcsk1*, and *sox2* expressed in low levels and only in a small portion of the cells regardless of the subpopulation, except for the highly expressed gene *mki67* (Figure S6). Together, this classification underscores the multifunctional nature of several genes and highlights how discrete gene subsets exhibit specific yet overlapping functional roles in GnRH neuron biology.

### 2.2 Single‐nuclei trajectory inference and functional analyses identify putative transcriptomic relationships among GnRH3 subpopulations

**FIGURE 3:**
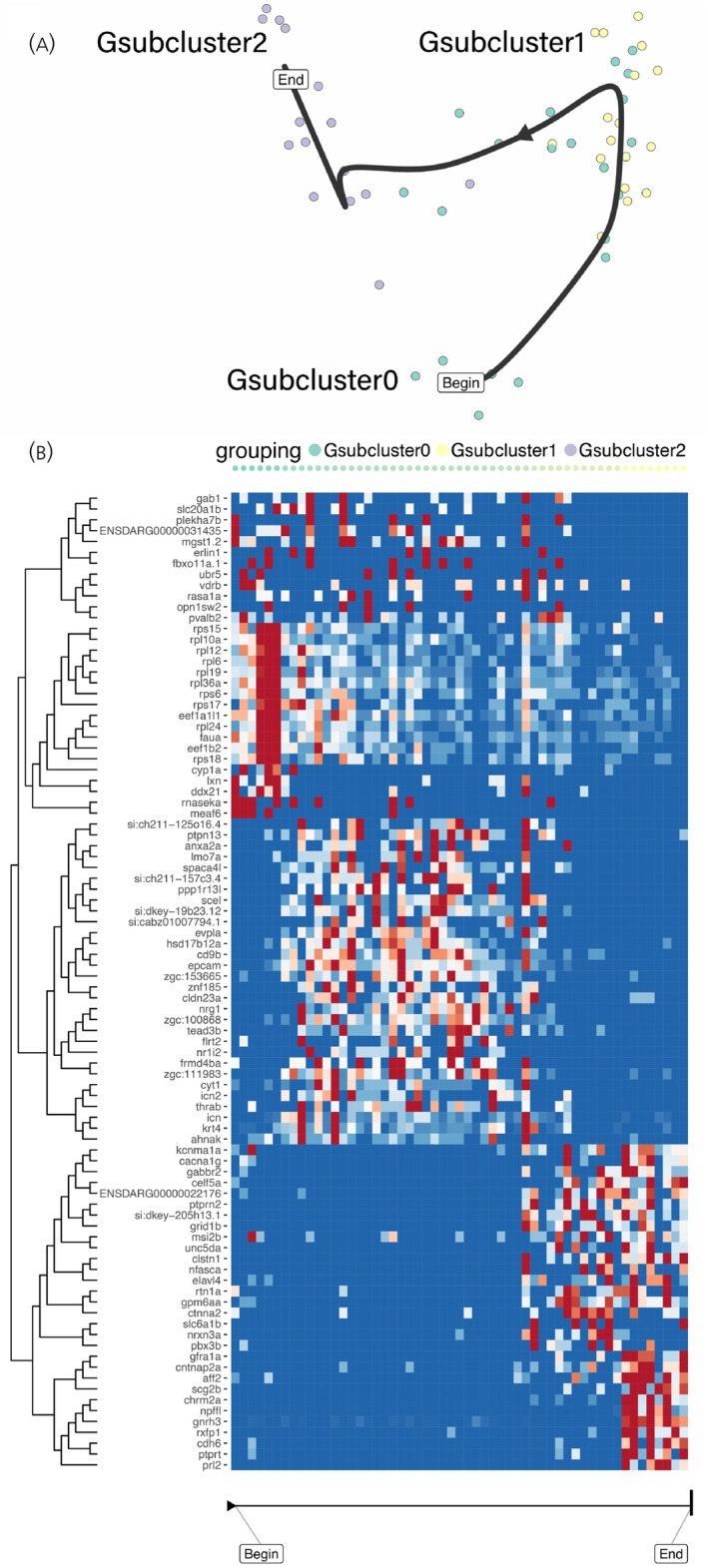
Single‐nuclei trajectory inference suggests a putative pseudotime continuum among GnRH3 subclusters. (A) Hypothetical pseudotime relationship inferred using Dynverse. (B) Heatmap of the top 90 differentially expressed genes (top 30 DEGs in each Gsubcluster) in the three Gsubclusters through pseudotime. Red and blue colors represent up‐regulation and down‐regulation, respectively.

Trajectory inference using the top 90 DEGs placed the three Gsubclusters along a pseudotime axis. Gsubcluster0 was enriched at earlier pseudotime, Gsubcluster1 occupied an intermediate pseudotime position, and Gsubcluster2 was enriched at later pseudotime (Figure 3A). We interpret this ordering as an exploratory representation of transcriptomic similarity among states, rather than definitive developmental stages or lineage transitions. The heatmap of the top 90 DEGs (Figure 3B) shows three dynamic expression patterns: one group of genes (e.g., *cyp1a*, *pvalb2*, and an array of ribosomal associated protein genes) is strongly upregulated at early pseudotime (Gsubcluster0), a second group peaks at intermediate pseudotime (Gsubcluster1), and a third group (e.g., *npffl* and *prl2*) increases toward late pseudotime (Gsubcluster2). These expression trends are consistent with transcriptomic differences across pseudotime from Gsubcluster0 to Gsubcluster2.

**FIGURE 4:**
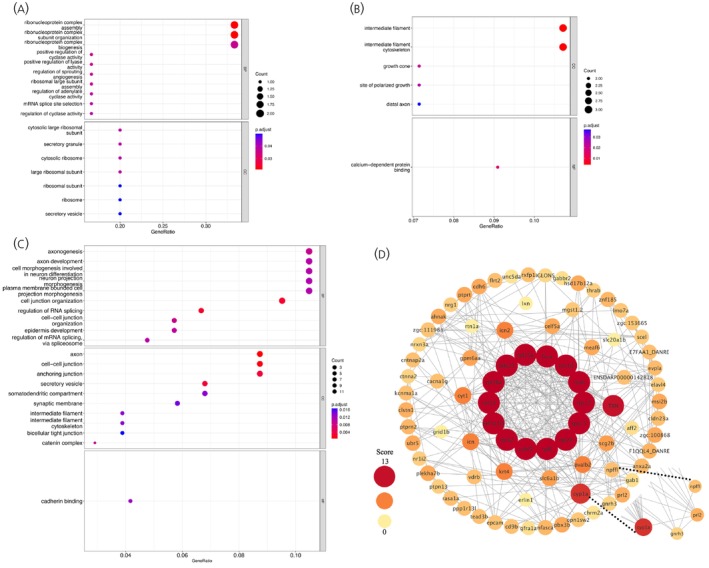
Gene ontology (GO) categories patterns and protein–protein interaction (PPI) networks of the differentially expressed genes (DEGs). (A) DEGs in Gsubcluster0 assigned to biological processes (BP) and cellular components (CC) terms. No DEG assigned to molecular functions (MF) categories. (B) DEGs in Gsubcluster1 assigned to CC and MF terms. No DEG assigned to BP categories. (C) DEGs in Gsubcluster2 assigned to BP, CC, and MF categories. (D) PPI networks of top 90 DEGs in three Gsubclusters (top 30 DEGs in each subcluster). Nodes and edges represent proteins and protein–protein associations, respectively. The intensity of the protein–protein interactions is represented by the color and size of dots.

Gene Ontology (GO) enrichment analysis of each Gsubcluster's DEGs revealed distinct functional profiles (Figure 4A–C). For Gsubcluster0, DEGs were significantly enriched in biological process (BP) terms related to ribonucleoprotein complex assembly, ribonucleoprotein complex subunit organization, and ribonucleoprotein complex biogenesis (Figure 4A). Corresponding cellular component (CC) terms included ribosomal subunit components. This enrichment is consistent with the high expression of ribosomal and translation‐related genes observed in Gsubcluster0. No molecular function (MF) terms were enriched for Gsubcluster0 DEGs. Gsubcluster1 DEGs were enriched in CC categories included intermediate filament and intermediate filament cytoskeleton, and in the MF‐calcium‐dependent protein binding (Figure 4B). No significant BP terms were found for Gsubcluster1. These GO categories suggest that intermediate cytoskeletal elements and calcium‐binding functions are prominent in this intermediate subcluster's gene set. For Gsubcluster2, DEGs showed enrichment in BP involved in neuron projection development and cell–cell junction organization, and in CC such as axon (Figure 4C). One MF term was also enriched. These categories align with the upregulated genes shown in Gsubcluster 2 (e.g., *npffl*, *prl2*), which may be associated with neuronal projection growth and adhesion.

The protein–protein interaction (PPI) relations among DEGs identified 14 genes, *rpl12*, *rps18*, *faua*, *rps6*, *rpl10a*, *rpl19*, *rps17*, *rpl36a*, *rpl24*, *eef1a1l1*, *rps15*, *zdbf2*, *rpl6*, and *ddx21*, which had the highest intensity of the protein–protein interactions (MCODE score = 13), contributing to the transcriptional process and ribosomal structures controlling metabolism and catabolism of amino acids (Figure 4D). However, it is not clear which genes are more significant and influence the others in GnRH3 neuron development and migration process; thus, we focused on GnRH3 in the PPI network to discover the relations within the PPI module including *gnrh3* (Figure 4D). We found that *npffl* and *prl2* directly interact with *gnrh3*. Furthermore, *prl2* interacts with *cyp1a* (MCODE score = 9.85); the latter is a gene known to exert a regulatory effect on multiple genes (Figure 4D).

### 2.3 Localization of selected markers from the different Gsubclusters

**FIGURE 5:**
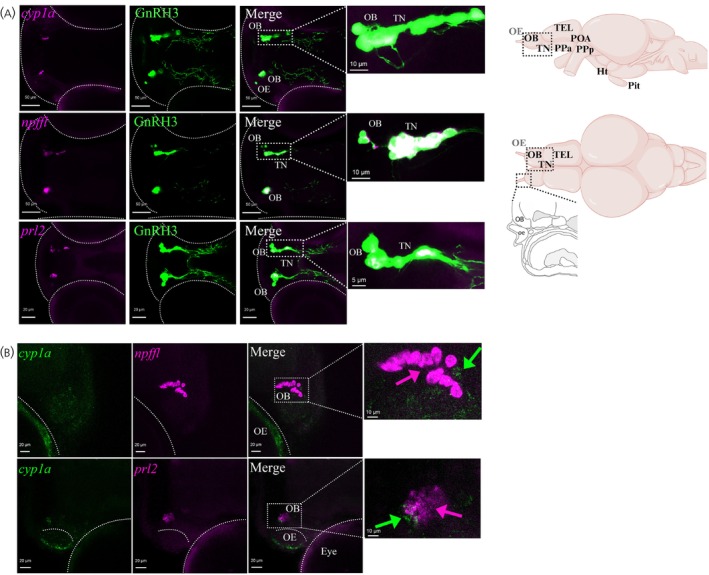
Spatial localization and co‐expression relationships of Gsubcluster‐specific markers in the GnRH3 system of 7 dpf zebrafish. (A) Whole‐mount ISH analysis of *Tg(gnrh3:EGFP)* larvae showing the distribution of Gsubcluster 0 and Gsubcluster 2 markers within the olfactory epithelium (OE), olfactory bulb (OB), and terminal nerve (TN) (*n* = 3, three sections imaged per fish). *Cyp1a*, *npffl*, and *prl2* mRNA are labeled in magenta, and GnRH3/GFP neurons are labeled in green. High‐magnification views (right panels; dashed boxes) highlight regions of signal overlap, revealing gene‐specific co‐localization within distinct GnRH3 neuronal subsets. Lateral and dorsal schematic views delineate the anatomical domains in which hybridization signals were detected. (B) Double *in situ* hybridization of wild‐type 7 dpf larvae examining the relationship between *cyp1a* (green) and either *npffl* or *prl2* (magenta) (*n* = 3, three sections imaged per fish). Enlarged merged views (right panels) demonstrate that *cyp1a* ^+^ cells (green arrows) are spatially adjacent but molecularly distinct from *npffl* ^+^ and *prl2* ^+^ cells (magenta arrows) in the OB/OE region. Corresponding anatomical schematics indicate the spatial context of detected signals. OE, olfactory epithelium; OB, olfactory bulb; TN, terminal nerve; TEL, telencephalon; POA, preoptic area; PPa/PPp, parvocellular preoptic nucleus (anterior/posterior); Ht, hypothalamus; Pit, pituitary. OB/OE outlines were adapted from https://zebrafishucl.org/forebrain‐regions/preglomerular‐complex.

Using whole‐mount ISH for *cyp1a*, *npffl*, and *prl2*, and IHC for *nrg1* on 7 dpf *Tg(gnrh3:EGFP)*, *cyp1a* was co‐localized with Gnrh3 in the olfactory placode (OP)/olfactory epithelium (OE) and olfactory bulbs (OB), while *npffl* and *prl2* from Gsubcluster 2 were found in the OB and terminal nerve (TN) (Figure 5A). The results corroborate the relative expression of these genes from the DEGs analysis of Gsubcluster0 (*cyp1a*: log2FC = 1.05, *p* = .0079) and Gsubcluster2 (*npffl*: log2FC = 4.84, *p* = 4.81E‐07 and *prl2*: log2FC = 2.29, *p* = 1.35E‐05) (Tables S5–S7). *Nrg1* (log2FC = 1.80, *p* = .0011) from Gsubcluster1 was not detected in GnRH3 neurons in 7 dpf *Tg(gnrh3:EGFP)*.

Double *ISH* of *cyp1a* with either *prl2* or *npffl* shows that *cyp1a* is not co‐expressed with *prl2* and *npffl* (Figure 5B). *Cyp1a* expression level is relatively low in the OB and seems to gradually decline along the migratory path (Figure 5).

### 2.4 Co‐expression of *npffl*, *prl2*, *cyp1a*, and *nrg1* in GnRH3 neurons in the adult brain

**FIGURE 6:**
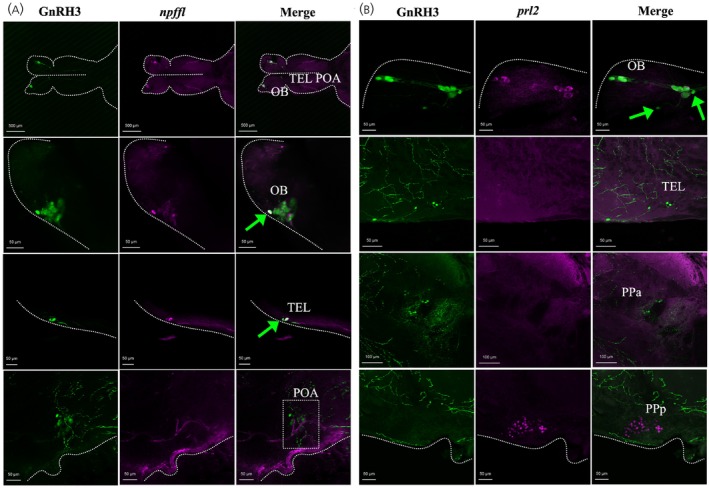
ISH of *npffl* (A) and *prl2* (B) distribution in mature female brain. (A) Superior view demonstrating the GnRH3 terminal nerve (green) and *npffl* (magenta) from the OB through the TEL (*n* = 3 female fish, three sections imaged per fish). All terminal nerve GnRH3 neurons (green arrows) co‐express *npffl* in the OB and TEL (sagittal view). POA GnRH3 neurons (white dashed box) in the PPp do not co‐express *npffl*. (B) Most OB *prl2* neurons (magenta), but not all (green arrow), co‐localize with GnRH3 neurons (green) (*n* = 3 female fish, three sections imaged per fish). GnRH3 neurons in the TEL and in the POA (PPa) do not express *prl2*. A large population of *prl2* expressing neurons that do not coexpress GnRH3, is found in the basal lateral hypothalamus.

Using ISH on adult female brain sections, we detected *npffl* mRNA in all OB and telencephalon (TEL) GnRH3 neurons but not in the preoptic area (POA) (Figure 6A).

*Prl2* was detected only in the larger GnRH3 neurons in the OB (Figure 6B). A novel large non‐GnRH3 population was detected in the parvocellular preoptic nucleus, posterior parts (PPp) (Figure 6B).

**FIGURE 7:**
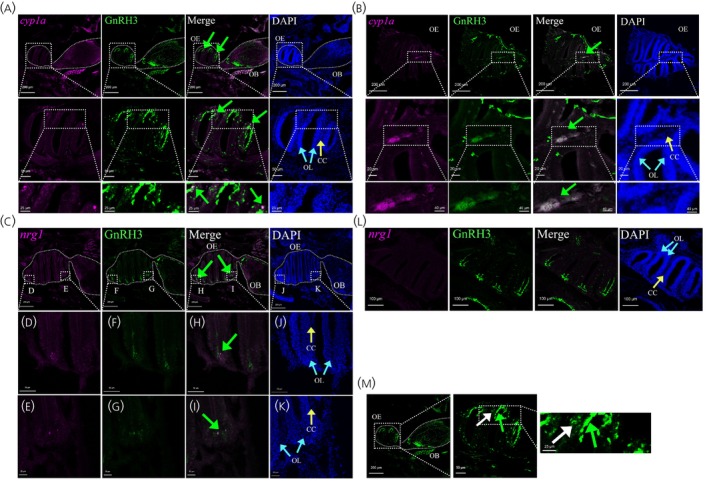
Detection of *cyp1a* and NRG1 in the OE and GFP‐intensity variation in OE GnRH3 neurons of mature zebrafish. Blue channel showing nuclear staining corresponding to the same field. ISH detection of *cyp1a* in mature (A) male and (B) female brain in the OE (*n* = 3 fish for each sex; three sections imaged per fish). *Cyp1a* expressing cells (magenta) are found in the olfactory lamellae (OL)/central core (CC) and co‐localize with some GnRH3 neurons (green). Higher‐magnification images of the regions delineated by dashed boxes are presented in the corresponding lower panels. (C) IHC of NRG1 in mature male brain in the OE (*n* = 3 male fish, three sections imaged per fish). NRG1 containing cells (magenta) are found in the OL and co‐localized with GnRH3 neurons (green). Higher‐magnification images of the regions delineated by dashed boxes are presented in the corresponding lower panels (D–K). (L) NRG1 was not detected in OE of mature female brain. (M) Varied GFP intensity in GnRH3 neurons in the OB/OE region of mature zebrafish regardless of image plane (*n* = 3 female fish, three sections imaged per fish). The blue and yellow arrows indicate olfactory lamellae (OL) and central core (CC), respectively. The green and white arrows indicate GnRH3 neurons exhibiting high and low GFP fluorescence intensity, respectively. Olfactory lamellae (OL), central core (CC).

*Cyp1a* mRNA was localized in GnRH3 neurons in the olfactory lamellae (OL) of the OE of adult males (Figure 7A) and the central core (CC) of female zebrafish (Figure 7B). NRG1 was co‐localized in separate GnRH3 neurons only in the OE‐OL of male zebrafish using IHC (Figure 7C). In female zebrafish, NRG1 was not detected (Figure 7L). The specificity of the NRG1 antibody was confirmed using immunostaining without the primary antibody (Figure S7). Differences in GFP intensity are notable among GnRH3 neurons in the OB/OE region of adult zebrafish (Figure 7M). Consistently, GnRH3 neurons in the OE region expressing *cyp1a* exhibited higher GFP intensity than those expressing *nrg1* (Figure 7).

## 3 DISCUSSION

In this study, three subpopulations of GnRH3 neurons, defined by highly expressed marker genes (*cyp1a*, *nrg1*, *npffl*, and *prl2*) were identified in 7 dpf larvae *Tg*(*GnRH3:GFP*) using snRNA‐Seq. These genes were selected for their prevalence in the different Gsubclusters and direct/indirect interactions with *gnrh3*.

*ISH* located GnRH3^cyp1a^ in the OE and the anterior part of the OB, GnRH3^npffl^ and GnRH3^prl2^ in the OB/TN, while GnRH3^nrg1^ cells were localized in the OE of adult zebrafish. Trajectory inference and GO enrichment analyses were consistent with a putative transcriptomic continuum among GnRH3‐associated states distributed from the OE to the TN. While the co‐expression of *cyp1a* and *nrg1* in Gnrh3 neurons is novel, *npffl* and *prl2* genes exhibit the strongest positive correlation with *gnrh3* as described in the zebrafish single‐cell atlas (DanioCell).^18^, ^19^

Abnormal or failed GnRH cell migration can lead to congenital hypogonadotropic hypogonadism (CHH) and infertility.^16^, ^20^ Various adhesion proteins, guidance molecules, and external cues guide the migrating cells. However, the molecules identified so far in these disorders only account for less than 50% of CHH cases, with the rest remaining to be revealed.^21^ *Npffl*, as one of the 32 unique genes in the merged PPI network analysis, interacts with important reproductive‐related factors like kiss and gpr74 (npffr), implying a role in reproduction.^22^ The exclusive *NPFF* presence in GnRH3 neurons in the OB, TN, and TEL has been previously described in larvae and adult zebrafish.^23^ The role of NPFF is controversial as it inhibits TN‐GnRH neurons firing in the dwarf gourami^24^ but is regulating behavioral motivation through coordinating olfactory and visual inputs in mature medaka.^25^ Yet, *npff* gene distribution in mammalian brain differs from that in fish, absent in the anterior brain but abundant in the hypothalamus, brain stem, and spinal cord.^26^

Unlike *prl*, which is expressed in the pituitary, *prl2* is a non‐mammalian isoform mainly expressed in the brain.^27^ The information regarding *prl2* in fish is scarce, and both the GnRH3^prl2^ neurons in the OB/TN and the PPp *prl2* populations warrant further investigation. These neurons may engage in unique neuropeptidergic signaling or coordinated secretion of multiple signaling factors, reinforcing the suggested modulating role of GnRH3^npffl/prl2^ neurons.^7^, ^28^

Cytochrome P450 1A is a crucial enzyme for metabolizing xenobiotics, including drugs and environmental toxins.^29^, ^30^, ^31^ Its functional significance in GnRH neurons remains unknown, although a previous study detected CYP1A immunoreactivity in the OB of 5 dph larvae and adult gilthead seabream, consistent with our finding.^32^ Thus, the absence of its expression in the TEL and POA in our study could be attributed to methodological or species‐specific differences. *Cyp1a* expression is modulated by steroid hormones in fish,^33^, ^34^, ^35^, ^36^ inferring a role in the control of reproduction. GnRH3^nrg1^ was only found in the OE of adult zebrafish, despite being detected in larvae via snRNA‐Seq, which may be caused by the low translation rate of this gene. *Nrg1* is crucial for neural development, promoting growth and differentiation of neurons and glial cells and plays a significant role in synaptic plasticity, development, and function.^37^, ^38^ NRG1 mediates neurotransmission in the adult brain of mice.^37^ The functional role of GnRH3^nrg1^ cells in the OE is yet to be revealed, and their actual presence in the OE should be better established in light of their scarcity and the very low prevalence of *gnrh3* transcript.

GnRH3‐OE neurons were identified as crypt sensory neurons, mediating sex pheromone signals essential for successful reproduction in zebrafish.^13^ Therefore, *cyp1a* and *nrg1* may function in both the developmental migration of GnRH3 neurons and reproductive processes in zebrafish.

Our results in adults suggest that sexual dimorphism may exist in the OE, not in the OB, TN, and TEL. Nrg1 immuno‐reactive GFP‐labeled neurons were found in the OE‐olfactory lamellae (OL) of mature males; none were detected in the OE of females. Additionally, *cyp1a* positive GFP‐labeled neurons were detected in OE‐OL of mature males, while they appeared as clusters of GFP‐labeled cells within the OE‐central core (CC) region of mature females.^39^ This sexual dimorphism may reflect differences in sexual‐related signals perception between the two sexes. Interestingly, *gnrh3* ^−/−^ males, but not females, performed poorly in terms of attractiveness to females and spawning success compared to WT males,^13^ underscoring the importance and functional difference of GnRH3 between male and female zebrafish.

Several previously reported genes associated with GnRH neuronal development and migration^16^, ^17^ exhibited variable expression levels and distributions across and within the Gsubclusters. Similarly, GFP intensity was higher in GnRH3^cyp1a^ compared with GnRH3^nrg1^. *Gnrh3* and *EGFP* showed heterogeneous expression within Gsubclusters, further underscoring the variability between individual *gnrh3* neurons. This may reflect heterogeneity in the transcriptional states of individual Gnrh3‐associated neurons, potentially related to developmental or migration‐associated programs.

In the pseudotime projection, Gsubcluster0 and Gsubcluster2 were set as the origin and end pseudotime positions owing to the high prevalence of notch receptor 2 (*notch2*) in Gsubcluster0, a proliferation marker in neuronal precursors^40^ and parvalbumin (*pvalb2*),^41^ which plays a central role in regulating neural plasticity and in shaping neural dynamics, while the high expression level of fibroblast growth factor 13a (*fgf13a*), which is involved in cell adhesion, neuronal path finding, and cell migration^42^ in Gsubcluster2. Genes like *cyp1a*, *pvalb2*, and some belonging to the *rpl/rps* family that characterize early development were found in Gsubcluster0. This subcluster showed increased function in the BP category such as ribonucleoprotein complex assembly, ribonucleoprotein complex subunit organization, and ribonucleoprotein complex biogenesis, which function in/before the early stage of GnRH3 neuron migration. As to Gsubcluster1, Notch1/ICN2 and NRG1 represent distinct, yet interconnected, signaling pathways that play essential roles in the development and function of the nervous system.^43^ The enrichment analysis of Gsubcluster1 identified categories related to the intermediate transcriptomic state of GnRH3 neuron migration such as intermediate filament in the CC category and calcium‐dependent protein binding in the MF category. The enriched terms of *fgf13a*, *npffl*, and *prl2* in Gsubcluster2 may be related to GnRH3 axon/neuron projection development and cell–cell junction in the BP category; axon, cell–cell junction, and anchoring junction in the CC category.

Gsubcluster0 and Gsubcluster1 exhibited higher *notch2* and *nrg1* mRNA prevalence. *Notch* signaling, involved in sustaining proliferation and repressing differentiation, triggers maturation from a progenitor state,^17^, ^44^ consistent with the earlier pseudotime position of Gsubcluster0 in our exploratory trajectory analysis.

It is important to note that promoter‐driven reporters may capture transient gene activity during development, as previously demonstrated for GnRH promoter transgenics in mammals.^45^ Accordingly, although trajectory inference suggests putative relationships among transcriptomic states in the present dataset, these results should be interpreted as hypothesis‐generating rather than definitive developmental lineage relationships. Future longitudinal approaches, such as genetic lineage tracing or time‐resolved imaging of GnRH3 neurons, will be required to directly test developmental transitions among these subpopulations.

The relatively small number of nuclei with detectable gnrh3 transcripts and the low nuclear gnrh3 counts should be interpreted in the context of both biological rarity, the relatively low abundance of EGFP/gnrh3 in the nuclei compared to the cytosol, and technical features of snRNA‐seq. GnRH3 neurons are an exceptionally rare population in 7 dpf larvae,^13^ and the 4307 nuclei obtained from the total of approximately 10,000 represent post‐library, post‐QC nuclei derived from the EGFP‐enriched FACS fraction. In addition, snRNA‐seq captures nuclear transcripts and may under‐detect low‐abundance or predominantly cytoplasmic mature mRNAs. Thus, nuclei derived from gnrh3‐promoter‐active neurons may show low or undetectable nuclear gnrh3 counts. We therefore used detectable gnrh3 expression as a conservative molecular anchor for exploratory subclustering. Importantly, the low transcripts and low abundance of GnRH3 nuclei have been localized in the olfactory epithelium, a region which is known to contain GnRH3 neurons and established to be in the developmental path of GnRH3 neurons of zebrafish and GnRH1 in other species, from the nasal placode to the brain.^13^, ^46^ Nevertheless, we acknowledge that low nuclear gnrh3 counts limit the strength of cluster identity assignment, particularly for Gsubcluster0 and Gsubcluster1, and our conclusions are therefore framed as transcriptomic states.

The cell–cell communication between GnRH3 neurons is crucial in facilitating effective migration towards their destination.^8^, ^12^ During their pause at the NFJ from 24 hpf to 4 dpf, GnRH3 neurons communicate with each other through glutamatergic synapses.^12^ Our snRNA‐Seq found that *glutamate receptor*, *ionotropic*, *delta 1b* (*grid1b*) is highly expressed in GnRH3^npffl/prl2^ neurons (Gsubcluster2). *Grid1b* is involved in glutamatergic synaptic transmission and modulation of chemical synaptic transmission^47^ and may play an important role in GnRH3 neurons' communication in the OB during ontogeny.

The origin of the zebrafish POA GnRH3 neurons, the diversity of subpopulations, migratory pathways, and interactions among various subpopulations are subjects of ongoing debate.^13^, ^17^ Laser ablation of GnRH3 neurons in the OB at 2 dpf resulted in a GnRH3‐less adult brain, inferring that both the POA‐hypothalamic and TN populations originate from the olfactory region.^10^ Early study suggested that the TN and hypophysiotropic GnRH3 neurons originate from developing cranial neural crest and anterior pituitary placode, respectively.^48^ However, it was subsequently shown that early zebrafish olfactory epithelial cell‐type heterogeneity is established by multiple fate‐restricted progenitors derived from the preplacodal ectoderm (PPE).^46^ A recent study suggests that GnRH3 neurons undergo bi‐directional migration, with one subpopulation migrating anteriorly to populate the OE, while the others migrate posteriorly reaching the hypothalamus.^13^ Another study suggested that the migrating GnRH3 neurons consist of large and small cell populations, in which the large cells comprise the TN ganglion populations and the small cells continue to migrate into the POA.^49^ Our results confirm the presence of small and large cells in the OB/TN, and reveal that *prl2* is not expressed in the small cells in the OB.

In summary, trajectory inference and GO analyses are consistent with a putative transcriptomic continuum among GnRH3‐associated states, with *Gnrh3* ^*cyp1a*^ neurons enriched in the OE‐associated state, *Gnrh3* ^*nrg1*^ neurons representing a distinct intermediate state, and *Gnrh3* ^*npffl/prl2*^ neurons corresponding to an OB/TN‐associated state. Whether these states represent sequential developmental transitions remains unclear and will require longitudinal validation.

**FIGURE 8:**
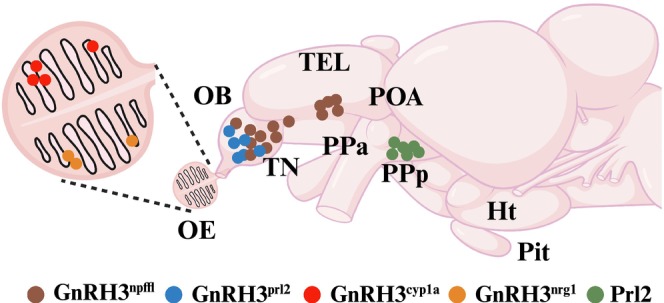
Graphical depiction of the localization of GnRH3^npffl^ (brown circles), GnRH3^prl2^ (blue circles), GnRH3^cyp1a^ (red circles), GnRH3^nrg1^ (orange circles), and *prl2* mRNA (green circles) in the brain of mature zebrafish. Created with BioRender.

The present study supports the presence of at least three molecularly distinct GnRH3‐associated subpopulations/states with differential transcriptomic characteristics in zebrafish. Single‐nuclei trajectory inference and GO enrichment analyses suggest putative relationships among these states and highlight candidate migration‐ or development‐associated programs. Moreover, our findings reveal expression variability within Gsubclusters, suggesting temporal or state‐dependent heterogeneity among GnRH3 neurons (Figure 8). Continued genetic and biochemical manipulations for the first revelation of identified marker genes promise valuable insights into the migration intrinsic mechanism of GnRH neurons during their ontogeny.

## 4 MATERIALS AND METHODS

### 4.1 Animal husbandry and maintenance

Adult zebrafish of Tübingen wild type (WT) and *Tg(gnrh3:EGFP)* lines were maintained in a recirculating system at 28°C with a day/night cycle of 14/10 h at the Institute of Marine and Environmental Technology (IMET). Commercial pellets (Gemma Micro 500) and brine shrimp were used to feed them twice daily. Zebrafish larvae for *in situ* hybridization chain reaction (HCR) were maintained in fish water prepared with 60 mg/L Instant Ocean salts at 28°C and fed once daily with paramecia after 4 days post‐fertilization (dpf). All animal experiments were performed following institutional guidelines and were approved by the Institutional Animal Care and Use Committee at the University of Maryland School of Medicine.

### 4.2 Cell dissociation and GFP‐labeled nuclei collection

GnRH3 neuronal nuclei were isolated from 7 dpf *Tg(gnrh3:EGFP)* zebrafish larvae, which has been widely used to visualize GnRH3 neurons *in vivo*, and GFP expression driven by the gnrh3 promoter has been shown to correspond closely with GnRH3 peptide or mRNA expression in previous studies.^8^, ^50^, ^51^ At this stage, GnRH3 neurons are distributed along their migration path. Nuclei preparation from whole larvae followed the protocol published elsewhere.^52^ 250 live larvae were euthanized by chilling on ice for 20 min, deyolked by vigorous pipetting with 200 μl tip using 3 mL swelling buffer (250 mM Sucrose, 10 mM Tris–HCL (pH 7.9), 10 mM MgCl2, and 1 mM EGTA). The mixture was passed through a 40 μM filter. The retained embryos were transferred to a tube containing 3.5 mL of cell lysis buffer (Nuclei EZ Lysis Buffer, Sigma) and incubated on ice for 30–40 min, during which the solution was passed through a 3‐mL syringe equipped with 22‐gauge needle every 5 min. The mixture was then centrifuged at 500*g* at 4°C for 5 min and the supernatant was collected twice and pooled supernatant was filtered through 20 μm filter and resuspended in 1 mL PBS + 1% BSA containing 200 units/mL protector RNase inhibitor (Millipore Sigma). Fluorescence‐activated nuclei sorting was performed using a BD FACS Aria II cell sorter equipped with a 488‐nm laser for excitation of GFP and 7‐AAD. Nuclei were first gated using forward‐ and side‐scatter properties to exclude debris, and doublets/aggregates were removed using FSC‐A versus FSC‐W pulse‐geometry parameters. Intact nuclei were identified by 7‐AAD fluorescence. Within the 7‐AAD‐positive singlet population, GFP‐positive nuclei were selected based on GFP fluorescence intensity. Autofluorescent events were identified using a V450 versus GFP plot and excluded from the final sorting gate. The final clean GFP‐positive gate was used for sorting. WT nuclei lacking GFP expression were used as negative controls to define background GFP fluorescence and establish the GFP gate. The gating workflow for WT controls and *Tg(gnrh3:EGFP)* samples is shown in Figure S8.

### 4.3 Library preparation and single‐nuclei RNA sequencing

Nuclei were isolated and libraries were prepared from approximately 10,000 nuclei using the 10 × Genomics Chromium platform according to the manufacturer's instructions. Libraries were sequenced on an Illumina NovaSeq 6000 system. Reads were aligned to the zebrafish reference genome (GRCz11), with protein‐coding genes retained and the green fluorescent protein (GFP) sequence included in the reference. Initial quality control, normalization, and downstream analyses were performed using standard pipelines. Detailed protocols and parameter settings are provided in Data S1.

### 4.4 Nuclei clustering

The generated Seurat data was subjected to dimensional reduction using principal component analysis (PCA) followed by uniform manifold approximation and projection (UMAP) using the first 30 principal components (PCs). *FindNeighbors* (dims = 1:30) function for defining “k‐nearest neighbors” among cells and then *FindClusters* (resolution = 0.2) function for finding the cell clusters were used.

Given transcript dropout in snRNA‐seq and the scarcity of GnRH3 neurons in 7 dpf larvae, we initially retained nuclei with detectable *gnrh3* transcripts (gnrh3 > 0) for exploratory subclustering. Reclustering was conducted as described above using Seurat object that only contains these nuclei followed by the above steps. Three subclusters (Gsubcluster0–2) were identified after reclustering.

### 4.5 Marker gene identification

Markers (differentially expressed genes, DEGs), *p* values and log2FC values were acquired for all detected genes, for each subcluster against the remaining subclusters was identified using Wilcoxon rank‐sum test in *FindAllMarkers* function in Seurat. Furthermore, the Zebrafish Information Network (ZFIN) was referenced for the nomenclature of the marker genes.

### 4.6 Trajectory inference

Differentially expressed genes among subclusters were used to generate hypothesis‐generating pseudotime relationships using Dynverse package.^53^ Top 90 pseudotime‐dependent genes in three subclusters were visualized using a heatmap reflecting the expression trends of these genes.

### 4.7 Function analysis

Gene ontology (GO) enrichment analysis of DEGs (|log2FC| > 1 and *p* < .05) from each subcluster was performed using clusterProfiler package 4.7.1 in RStudio. In addition, protein–protein interaction (PPI) networks of top 90 DEGs in three subclusters were constructed using STRING database and visualized in Cytoscape 3.10.2 using Molecular Complex Detection (MCODE) analysis method.^54^ *Cyp1a* in Gsubcluster0, *nrg1* in Gsubcluster1, *npffl* and *prl2* in Gsubcluster 2 were selected for the following analysis.

### 4.8 Whole mount *in situ* hybridization chain reaction

Hybridization chain reaction (HCR) reagents (probes, hairpins, and buffers) used in this study were purchased from Molecular Instruments. The staining of 7 dpf WT and *Tg(gnrh3:EGFP)* larvae followed the manufacturer's “HCR™ RNA‐FISH protocol for whole‐mount zebrafish embryos and larvae.”^55^ Probes were prepared based on gene accession: *npffl* (ENSDARG00000045016), *prl2* (ENSDARG00000018744), and *cyp1a* (ENSDARG00000098315). Confocal imaging was performed on larval heads mounted in 1% low‐melting agarose (see Data S2 for detailed protocol).

### 4.9 *In situ* hybridization on adult brain sections

To prepare cryosections of adult male and female brains (*n* = 3 fish for each sex; three sections imaged per fish), the upper (superior) craniums were removed, and exposed brains were fixed in 4% PFA overnight at 4°C. Intact brains were dissected out and were immersed in 30% sucrose in PBS until the tissue sank. OCT frozen 15 μm section were subjected to HCR RNA‐FISH,^55^ with detailed protocols provided in Data S3. Confocal imaging was performed on slides mounted with antifade reagent containing DAPI.

### 4.10 Immunohistochemistry on adult brain sections

Fixation, cryopreservation, and sectioning of whole head of adult male and female zebrafish heads (*n* = 3 fish for each sex; three sections imaged per fish) were decalcified for 4 days with 0.5M EDTA (pH 8.0) at 4°C between fixation and sucrose. The brains were mounted and cryosectioned sequentially at 15 μm thickness to preserve tissue architecture.

Air dried sections were fixed in pre‐chilled methanol (−20°C, 2 min). Following three PBS washes, slides were blocked in 1XPBS, 3% normal goat serum, 3% BSA, and 0.3% Triton X‐100 for 1 h followed by overnight incubation at 4°C with Rabbit anti‐zebrafish Nrg1 (Antibodies‐Online Cat# ABIN734828, RRID:AB_11206812), diluted 1:100 in PBS, 3% BSA, and 0.3% Triton X‐100. After four washes in PBST each section was incubated with goat anti‐rabbit secondary antibodies Cy3 (Thermo Fisher Scientific) diluted 1:1000 for 1 h, washed, and mounted with antifading mounting medium before imaging.

### 4.11 Confocal imaging

Confocal images of transversal view of heads of zebrafish larvae and sagittal view of adult zebrafish brain were acquired to detect marker genes and GnRH3 (EGFP) signals using Leica SP8 confocal microscope (Leica Microsystems). Photoshop (Adobe) and Fiji^56^ were used to process the images.

## AUTHOR CONTRIBUTIONS

**Xiaoxuan Fan:** Methodology; writing – review and editing; resources; visualization. **Yonathan Zohar:** Supervision; project administration; writing – review and editing; resources; funding acquisition. **Nilli Zmora:** Conceptualization; methodology; data curation; investigation; validation; formal analysis; supervision; funding acquisition; project administration; resources; writing – review and editing; writing – original draft. **Yalong Sun:** Conceptualization; methodology; software; validation; formal analysis; visualization; writing – original draft; data curation; investigation; writing – review and editing. **Matan Golan:** Conceptualization; methodology; investigation; supervision; project administration; writing – review and editing.

## CONFLICT OF INTEREST STATEMENT

The authors declare no conflict of interest.

## Supporting information


**Data S1.** Library preparation and single‐nuclei RNA sequencing, Zebrafish genome rebuilding and alignment, and initial data processing.
**Data S2.** Whole mount *in situ* hybridization chain reaction (HCR).
**Data S3.** *In situ* hybridization on adult brain sections.
**Table S1.** Top 30 differentiated marker genes of cluster0.
**Table S2.** Top 30 differentiated marker genes of cluster1.
**Table S3.** Top 30 differentiated marker genes of cluster2.
**Table S4.** Top 30 differentiated marker genes of cluster3.
**Table S5.** Top 30 differentiated marker genes of Gsubcluster0.
**Table S6.** Top 30 differentiated marker genes of Gsubcluster1.
**Table S7.** Top 30 differentiated marker genes of Gsubcluster2.
**Figure S1.** Relative expression and distribution of top 10 differentiated marker genes of cluster0.
**Figure S2.** Relative expression and distribution of top 10 differentiated marker genes of cluster1.
**Figure S3.** Relative expression and distribution of top 10 differentiated marker genes of cluster2.
**Figure S4.** Relative expression and distribution of top 10 differentiated marker genes of cluster3.
**Figure S5.** UpSet plot illustrating the functional categorization of 22 genes in zebrafish GnRH3 neurons across five categories: GnRH neuron development (Dev), migration (Mig), regulation of GnRH secretion/function (Reg), axon guidance/synaptic connectivity (Guid), and Kallmann syndrome/congenital hypogonadotropic hypogonadism (KS/CHH). The plot presents both the total number of genes assigned to each category (left) and the sizes of their specific intersections (top), revealing predominant and unique patterns of functional overlap among gene subsets.
**Figure S6.** Relative expression and distribution of specific genes involved in GnRH neuronal development and migration exhibit variable expression levels and distribution across different Gsubclusters.
**Figure S7.** Validation of NRG1 antibody specificity. Immunostaining was performed on mature male zebrafish in the absence of the primary NRG1 antibody to evaluate non‐specific background signals. No detectable magenta fluorescence was observed under these conditions, confirming that the secondary antibody and imaging settings did not generate non‐specific staining. These results verify the specificity of the NRG1 primary antibody used in Figure 7B. Green and blue channels showing GnRH3 and nuclear staining corresponding to the same field, respectively.
**Figure S8.** FACS gating strategy and WT negative controls for enrichment of clean EGFP‐positive nuclei from *Tg(gnrh3:EGFP)* zebrafish larvae. (A) FACS gating of 7 dpf WT nuclei stained with 7‐AAD. WT nuclei lacking GFP expression were used to define the background GFP fluorescence and establish the GFP‐positive gate. Only a minimal fraction of WT events fell within the DP/clean GFP‐positive gate. (B) FACS gating of 3 dpf WT nuclei stained with 7‐AAD, providing an additional GFP‐negative control for background fluorescence. (C) FACS gating of 7 dpf *Tg(gnrh3:EGFP)* nuclei. Events were first gated based on FSC‐A versus SSC‐A to exclude debris, followed by FSC‐A versus FSC‐W gating to enrich singlets. Within the singlet population, 7‐AAD‐positive/GFP‐positive events were selected using the double positive (DP) gate. A subsequent V450 versus GFP plot was used to distinguish auto‐fluorescent events from clean GFP‐positive nuclei. The auto‐fluorescence gate was excluded, and the clean GFP‐positive gate was used for sorting. The final clean GFP‐positive gate was positioned away from the auto‐fluorescence gate and used for downstream collection. FSC‐A, forward scatter area; SSC‐A, side scatter area; FSC‐W, forward scatter width; V450‐A, V450 fluorescence area.

## Data Availability

The data that support the findings of this study are openly available in GEO at https://www.ncbi.nlm.nih.gov/geo/query/acc.cgi?acc=GSE309816, reference number GSE309816. The snRNA‐seq datasets generated in this study have been deposited at the NCBI's Gene Expression Omnibus under accession number GSE309816. The datasets generated during this study are currently under private access. They will be made publicly available in GEO immediately upon acceptance and publication of the manuscript, superseding the current embargo date (July 30, 2026). For peer review, the data may be accessed using the secure token ankhocqejzqtnwr through the GEO private review link: https://www.ncbi.nlm.nih.gov/geo/query/acc.cgi?acc=GSE309816. No custom code was generated for this study. All analyses were conducted using publicly available software packages. Further requests are available from the corresponding authors upon reasonable request.
