# Supplementary material for: Single‐nuclei RNA sequencing reveals heterogeneity within developing GnRH3 neurons in zebrafish
Source: J Neuroendocrinol. 2026 Jul 7;38(7):e70230. doi: 10.1111/jne.70230 (PMC13342788; doi:10.1111/jne.70230)
Supplement: Supplementary file 1 — Data S1. Library preparation and single‐nuclei RNA sequencing, Zebrafish genome rebuilding and alignment, and initial data processing. Data S2. Whole mount in situ hybridization chain reaction (HCR). Data S3. In situ hybridization on adult brain sections. Table S1. Top 30 differentiated marker genes of cluster0. Table S2. Top 30 differentiated marker genes of cluster1. Table S3. Top 30 differentiated marker genes of cluster2. Table S4. Top 30 differentiated marker genes of cluster3. Table S5. Top 30 differentiated marker genes of Gsubcluster0. Table S6. Top 30 differentiated marker genes of Gsubcluster1. Table S7. Top 30 differentiated marker genes of Gsubcluster2. Figure S1. Relative expression and distribution of top 10 differentiated marker genes of cluster0. Figure S2. Relative expression and distribution of top 10 differentiated marker genes of cluster1. Figure S3. Relative expression and distribution of top 10 differentiated marker genes of cluster2. Figure S4. Relative expression and distribution of top 10 differentiated marker genes of cluster3. Figure S5. UpSet plot illustrating the functional categorization of 22 genes in zebrafish GnRH3 neurons across five categories: GnRH neuron development (Dev), migration (Mig), regulation of GnRH secretion/function (Reg), axon guidance/synaptic connectivity (Guid), and Kallmann syndrome/congenital hypogonadotropic hypogonadism (KS/CHH). The plot presents both the total number of genes assigned to each category (left) and the sizes of their specific intersections (top), revealing predominant and unique patterns of functional overlap among gene subsets. Figure S6. Relative expression and distribution of specific genes involved in GnRH neuronal development and migration exhibit variable expression levels and distribution across different Gsubclusters. Figure S7. Validation of NRG1 antibody specificity. Immunostaining was performed on mature male zebrafish in the absence of the primary NRG1 antibody to evaluate non‐specif [file JNE-38-e70230-s001.docx]

**Single-Nuclei RNA Sequencing Reveals Heterogeneity Within Developing GnRH3 Neurons in Zebrafish**

Yalong Sun^1^, Matan Golan^2^, Yonathan Zohar^1^, Xiaoxuan Fan^3^ & Nilli Zmora^1*^

^1^Institute of Marine & Environmental Technology, Department of Marine Biotechnology, University of Maryland Baltimore County, Baltimore, MD 21202, USA

^2^Department of Animal Sciences, The Robert H. Smith Faculty of Agriculture, Food, and Environment, The Hebrew University of Jerusalem, Rehovot 76100, Israel

^3^Department of Microbiology and Immunology, University of Maryland Baltimore, Baltimore, MD 21201, USA

Correspondence: Nilli Zmora, PhD, Institute of Marine & Environmental Technology, Department of Marine Biotechnology, University of Maryland Baltimore County, Baltimore, MD 21202, USA. Email: [nzmora@umbc.edu](mailto:nzmora@umbc.edu).

ORCID

Yalong Sun https://orcid.org/0009-0001-8713-3441

Matan Golan https://orcid.org/0000-0003-4861-5912

Yonathan Zohar https://orcid.org/0000-0003-1092-3557

Nilli Zmora <https://orcid.org/0000-0003-1324-2458>

Short Title: Heterogeneity within developing GnRH3 neurons

Keywords: single-nuclei RNA sequencing, gonadotropin releasing hormone neurons, migration, heterogeneity

**Supplementary Information**

**Supplementary information 1.**

- 1. Library preparation and single-nuclei RNA sequencing

Single-nuclei cDNA library preparation and sequencing were conducted at the Genomics Resource Center core facility, Institute for Genome Sciences, University of Maryland School of Medicine (Baltimore, MD, USA). Single-nuclei RNA sequencing (snRNA-Seq) cDNA library was prepared from 10,000 nuclei using Chromium Next GEM Single Cell 3' GEM, Library & Gel Bead Kit v3.1 (10×Genomics, Pleasanton, CA, USA) on the 10×Genomics Chromium Controller according to the manufacturer’s manual. cDNAs were barcoded followed by library sequenced on the Illumina NovaSeq6000 platform with 100-bp paired-end reads at a depth of 77,538 mean reads per nucleus.

- 1. Zebrafish genome rebuilding and alignment

After the gene annotation of the zebrafish genome- GRCz11/danRer11 (Ensembl release 106; GCA_000002035.4) was filtered using *cellranger mkgtf* command (--*attribute=gene_biotype:protein_coding*, only protein-coding genes were included), green fluorescent protein (GFP) sequence and information (gene_id "GFP"; transcript_id "GFP"; gene_name "GFP"; gene_biotype "protein_coding";) were added to the zebrafish genome as reference using *cellranger mkref* command for the following analysis in 10×Genomics Cell Ranger software v7.1.0 (10×Genomics, Pleasanton, CA, USA).

Genome alignment using STAR, preliminary filtering for reads, barcode and UMI counting were performed and then a feature-barcode matrix (gene-by-cell expression count matrix) was generated using *cellranger_count* pipeline with default parameters.

- 1. Initial data processing

Using the Seurat v4.3.0 R package in an R v4.2.3 environment, cells with less than 200 or more than 2500 detected genes per cell and more than 10% mitochondrial genes per cell were excluded.

The filtered single-cell data sets were normalized using *SCTransform* functions (*variable.features.n = 5000*, using 5,000 features as variable features after ranking by residual variance) and top 5,000 highly variable genes were selected from the Seurat analysis. In addition, the percentages of mitochondrial genes and calculated cell cycle scores for each cell were included as variables to regress the data.

**Supplementary information 2. Whole mount *in situ* hybridization chain reaction (HCR)**

Whole-mount HCR was conducted on 7 dpf WT and *Tg(gnrh3:EGFP)* larvae. Yolks were removed by trituration in buffer (55 mM NaCl, 3.6 mM KCl, 1.25 mM NaHCO₃). Samples were fixed in 4% paraformaldehyde (PFA) for 24 h at 4 °C, washed in PBS, and cleared in 3% H₂O₂ in 0.5% KOH until pigmentation disappeared. Larvae were dehydrated and permeabilized through a methanol (MeOH) series and stored at −20 °C overnight.

Larvae were rehydrated through a graded MeOH in PBST series (75%, 50%, 25%) and permeabilized in 30 µg/mL proteinase K for 30 min at RT. After two PBST washes, samples were post-fixed in 4% PFA for 20 min and washed five times in PBST. Pre-hybridization was performed in 500 μL of hybridization buffer for 30 min at 37 °C, followed by overnight hybridization at 37 °C with 2 pmol of each probe in 500 µL hybridization buffer.

Post-hybridization washes followed the manufacturer’s instructions. Signal amplification was carried out overnight at RT in amplification buffer containing 30 pmol each of hairpins h1 and h2. After final washes in DAPI-containing buffer, larval heads were dissected and mounted in 1% low-melting agarose for confocal imaging.

**Supplementary information 3. *In situ* hybridization on adult brain sections**

*In situ* hybridization was performed using the Hybridization Chain Reaction (HCR) RNA-FISH method . Tissue sections were fixed briefly in 4% paraformaldehyde and dehydrated through graded ethanol. Sections were treated with proteinase K (10 μg/mL, 37°C, 10 min), followed by pre-hybridization in hybridization buffer (37°C, 60 min). Probes (0.4 pmol per slide) were hybridized overnight at 65°C. After stringent washes in SSCT buffer, sections underwent amplification with snap-cooled fluorescent hairpin solutions (6 pmol each of h1 and h2) overnight at room temperature. Following final washes, slides were mounted with antifade reagent containing DAPI and covered with coverslips.

**Supplementary Table 1**: Top 30 differentiated marker genes of cluster0

| Gene | p_val | log2FC | Accession Number |
| --- | --- | --- | --- |
| *grid1b* | 1.19E-284 | 1.89164934 | ENSDARG00000044161 |
| *pbx3b* | 2.93E-244 | 1.76017772 | ENSDARG00000013615 |
| *elavl4* | 0 | 1.75669737 | ENSDARG00000045639 |
| *gpm6aa* | 0 | 1.75335398 | ENSDARG00000055455 |
| *nrxn1a* | 0 | 1.69201478 | ENSDARG00000061647 |
| *nlgn1* | 0 | 1.61940411 | ENSDARG00000077710 |
| *stmn1b* | 0 | 1.59058152 | ENSDARG00000033655 |
| *nrxn2a* | 0 | 1.559747 | ENSDARG00000061454 |
| *rbfox1* | 4.29E-295 | 1.5576792 | ENSDARG00000014746 |
| *ptmaa* | 2.38E-215 | 1.48902553 | ENSDARG00000021113 |
| *marcksl1b* | 9.80E-300 | 1.44044546 | ENSDARG00000035715 |
| *nrxn3a* | 1.56E-234 | 1.41131263 | ENSDARG00000043746 |
| *iglon5* | 0 | 1.38408725 | ENSDARG00000022176 |
| *asic2* | 1.03E-269 | 1.38316814 | ENSDARG00000006849 |
| *syt1a* | 0 | 1.30180446 | ENSDARG00000030614 |
| *unc5a* | 1.68E-273 | 1.29138496 | ENSDARG00000099034 |
| *pcdh7b* | 3.05E-127 | 1.28981149 | ENSDARG00000060610 |
| *elavl3* | 0 | 1.26806033 | ENSDARG00000014420 |
| *sv2a* | 0 | 1.18968493 | ENSDARG00000059945 |
| *aplp1* | 0 | 1.18233715 | ENSDARG00000098368 |
| *celf5a* | 1.04E-304 | 1.18091085 | ENSDARG00000071375 |
| *sncb* | 0 | 1.15813422 | ENSDARG00000104945 |
| *cdh4* | 6.83E-242 | 1.15563013 | ENSDARG00000015002 |
| *rbfox3a* | 2.55E-150 | 1.14940605 | ENSDARG00000010083 |
| *nova2* | 3.18E-305 | 1.14767287 | ENSDARG00000017673 |
| *ncam2* | 8.01E-163 | 1.14262288 | ENSDARG00000017466 |
| *tenm2* | 4.48E-204 | 1.12659541 | ENSDARG00000037122 |
| *stx1b* | 0 | 1.12197831 | ENSDARG00000000503 |
| *gpm6ab* | 3.02E-299 | 1.11181172 | ENSDARG00000004621 |
| *adgrl1a* | 1.67E-250 | 1.0827877 | ENSDARG00000089292 |

Supplementary Table 2: Top 30 differentiated marker genes of cluster1

| Gene | p_val | log2FC | Accession Number |
| --- | --- | --- | --- |
| *icn* | 8.49E-179 | 2.27097018 | ENSDARG00000009978 |
| *epcam* | 0 | 1.83204056 | ENSDARG00000040534 |
| *krt4* | 6.17E-190 | 1.75073634 | ENSDARG00000017624 |
| *aqp3a* | 2.51E-233 | 1.3411421 | ENSDARG00000003808 |
| *agr1* | 3.13E-155 | 1.30524362 | ENSDARG00000060682 |
| *apoeb* | 7.58E-78 | 1.25557873 | ENSDARG00000040295 |
| *zgc:153665* | 4.64E-188 | 1.23875684 | ENSDARG00000043442 |
| *cyt1* | 9.06E-158 | 1.23467936 | ENSDARG00000092947 |
| *krt91* | 1.19E-102 | 1.16900872 | ENSDARG00000036830 |
| *cldnb* | 1.32E-226 | 1.15134499 | ENSDARG00000009544 |
| *krt1-19d* | 1.52E-71 | 1.14969296 | ENSDARG00000023082 |
| *eppk1* | 9.42E-168 | 1.12445117 | ENSDARG00000096359 |
| *cldni* | 2.53E-104 | 1.12118327 | ENSDARG00000054616 |
| *ppl* | 3.02E-141 | 1.1021307 | ENSDARG00000101043 |
| *zgc:100868* | 7.94E-106 | 1.08251713 | ENSDARG00000004748 |
| *zgc:111983* | 2.13E-118 | 1.04521775 | ENSDARG00000006588 |
| *s100a10b* | 7.33E-185 | 1.02474188 | ENSDARG00000025254 |
| *rhbg* | 1.60E-128 | 1.02061285 | ENSDARG00000009018 |
| *cd9b* | 3.51E-184 | 0.99926274 | ENSDARG00000016691 |
| *cdh1* | 2.47E-250 | 0.98988088 | ENSDARG00000102750 |
| *si:dkey-19b23.12* | 3.04E-127 | 0.97757233 | ENSDARG00000100712 |
| *cldne* | 1.59E-118 | 0.96680051 | ENSDARG00000043128 |
| *hsd17b12a* | 2.25E-152 | 0.94447734 | ENSDARG00000015709 |
| *si:dkey-87o1.2* | 1.40E-158 | 0.94084747 | ENSDARG00000095796 |
| *cfl1l* | 4.64E-233 | 0.93174946 | ENSDARG00000012972 |
| *krt92* | 6.09E-55 | 0.90091996 | ENSDARG00000036834 |
| *zgc:165423* | 3.17E-68 | 0.88790299 | ENSDARG00000052905 |
| *ahnak* | 1.02E-119 | 0.84109858 | ENSDARG00000061764 |
| *ptprfb* | 2.92E-119 | 0.79800312 | ENSDARG00000005754 |
| *evplb* | 3.35E-101 | 0.75420072 | ENSDARG00000103459 |

Supplementary Table 3: Top 30 differentiated marker genes of cluster2

| Gene | p_val | log2FC | Accession Number |
| --- | --- | --- | --- |
| *fthl27* | 0 | 4.33379399 | ENSDARG00000031776 |
| *fthl28* | 0 | 4.1913648 | ENSDARG00000076221 |
| *hbbe1.1* | 0 | 3.84545312 | ENSDARG00000113599 |
| *sqstm1* | 0 | 3.5125315 | ENSDARG00000075014 |
| *fthl31* | 0 | 3.40738597 | ENSDARG00000094210 |
| *hsp90aa1.2* | 0 | 2.72890774 | ENSDARG00000024746 |
| *hsp70.3* | 0 | 2.67146779 | ENSDARG00000021924 |
| *ubb* | 0 | 2.50154794 | ENSDARG00000002369 |
| *hbae3* | 3.76E-299 | 2.47096553 | ENSDARG00000079305 |
| *hsp70.2* | 0 | 2.40024725 | ENSDARG00000092362 |
| *hbbe2* | 0 | 2.20257865 | ENSDARG00000045143 |
| *hbae1.3.1* | 6.31E-284 | 2.20050142 | ENSDARG00000089124 |
| *fth1a* | 0 | 2.187532 | ENSDARG00000015551 |
| *hspa8* | 9.29E-255 | 1.83651452 | ENSDARG00000037403 |
| *rplp0* | 1.58E-258 | 1.81871913 | ENSDARG00000051783 |
| *txn* | 0 | 1.79783588 | ENSDARG00000031435 |
| *fthl30* | 0 | 1.71262316 | ENSDARG00000077360 |
| *hbae1.3* | 2.81E-295 | 1.64566809 | ENSDARG00000089124 |
| *hbae1.1* | 0 | 1.63970001 | ENSDARG00000089124 |
| *creg1* | 0 | 1.62576837 | ENSDARG00000105154 |
| *ccng1* | 0 | 1.60822656 | ENSDARG00000076667 |
| *psmb7* | 0 | 1.58778012 | ENSDARG00000037962 |
| *eef1b2* | 1.10E-221 | 1.54445578 | ENSDARG00000044521 |
| *eif2s2* | 0 | 1.52303142 | ENSDARG00000053047 |
| *tpt1* | 2.75E-216 | 1.50152547 | ENSDARG00000092693 |
| *abcf1* | 0 | 1.49301204 | ENSDARG00000031795 |
| *ubb* | 0 | 1.48302239 | ENSDARG00000093313 |
| *mgst3b* | 1.69E-229 | 1.42510261 | ENSDARG00000033364 |
| *blvrb* | 0 | 1.37396713 | ENSDARG00000096829 |
| *erh* | 0 | 1.3739109 | ENSDARG00000032866 |

Supplementary Table 4: Top 30 differentiated marker genes of cluster3

| Gene | p_val | log2FC | Accession Number |
| --- | --- | --- | --- |
| *rbp4* | 1.36E-285 | 1.99117314 | ENSDARG00000101199 |
| *plpp3* | 0 | 1.63849011 | ENSDARG00000059933 |
| *si:ch211-251b21.1* | 3.67E-228 | 1.54801466 | ENSDARG00000007275 |
| *slc7a8a* | 3.67E-92 | 1.45800058 | ENSDARG00000075831 |
| *lpl* | 1.42E-231 | 1.44456619 | ENSDARG00000087697 |
| *col5a1* | 0 | 1.36363258 | ENSDARG00000012593 |
| *cyp1c1* | 7.13E-267 | 1.28790997 | ENSDARG00000101195 |
| *igfbp5b* | 7.05E-115 | 1.27151974 | ENSDARG00000025348 |
| *ccl25b* | 1.05E-59 | 1.22037519 | ENSDARG00000070873 |
| *rhag* | 1.52E-98 | 1.11143695 | ENSDARG00000019253 |
| *cd81a* | 4.06E-146 | 1.11078702 | ENSDARG00000036080 |
| *col1a1a* | 4.67E-206 | 1.11010455 | ENSDARG00000012405 |
| *col1a1b* | 1.50E-217 | 1.10236518 | ENSDARG00000035809 |
| *pmp22a* | 2.59E-293 | 1.08630994 | ENSDARG00000105223 |
| *ccnd2a* | 3.89E-124 | 1.0805199 | ENSDARG00000051748 |
| *si:dkey-151g10.6* | 2.03E-104 | 1.07391998 | ENSDARG00000092807 |
| *col1a2* | 1.21E-206 | 1.07090527 | ENSDARG00000020007 |
| *fstl1b* | 1.21E-187 | 1.03719241 | ENSDARG00000039576 |
| *cxcl12a* | 2.40E-89 | 1.02370467 | ENSDARG00000037116 |
| *rps4x* | 8.09E-81 | 1.01509603 | ENSDARG00000014690 |
| *rpl39* | 1.05E-87 | 1.00273173 | ENSDARG00000036316 |
| *Rps17* | 2.24E-81 | 0.98547971 | ENSDARG00000046157 |
| *zgc:153704* | 1.95E-193 | 0.98411048 | ENSDARG00000045979 |
| *kcnq5a* | 3.17E-22 | 0.97733648 | ENSDARG00000069954 |
| *rpl8* | 1.46E-86 | 0.97499726 | ENSDARG00000014867 |
| *rpl9* | 1.95E-86 | 0.96240463 | ENSDARG00000037350 |
| *rpl7* | 1.39E-81 | 0.96154586 | ENSDARG00000007320 |
| *rps15a* | 2.85E-85 | 0.96150407 | ENSDARG00000010160 |
| *LO018188.1* | 5.91E-180 | 0.95719057 | ENSDARG00000106873 |
| *igfbp1b* | 2.16E-29 | 0.95649194 | ENSDARG00000038666 |

**Supplementary Figure 1**: Relative expression and distribution of top 10 differentiated marker genes of cluster0.

**Supplementary Figure 2**: Relative expression and distribution of top 10 differentiated marker genes of cluster1.

**Supplementary Figure 3**: Relative expression and distribution of top 10 differentiated marker genes of cluster2.

**Supplementary Figure 4**: Relative expression and distribution of top 10 differentiated marker genes of cluster3.

Supplementary Table 5: Top 30 differentiated marker genes of Gsubcluster0

| Gene | p_val | log2FC | Accession Number |
| --- | --- | --- | --- |
| *cyp1a* | 0.00791234 | 1.052088511 | ENSDARG00000098315 |
| *rpl6* | 0.00310455 | 1.017921908 | ENSDARG00000058451 |
| *rpl10a* | 0.00056161 | 0.959358016 | ENSDARG00000042905 |
| *pvalb2* | 0.00177046 | 0.958420896 | ENSDARG00000002768 |
| *opn1sw2* | 0.00472047 | 0.932885804 | ENSDARG00000017274 |
| *ddx21* | 0.00039141 | 0.819427754 | ENSDARG00000063626 |
| *rps6* | 0.00197999 | 0.817135943 | ENSDARG00000019778 |
| *rps18* | 0.00163821 | 0.800691192 | ENSDARG00000100392 |
| *rpl24* | 0.00157844 | 0.797266104 | ENSDARG00000099104 |
| *rpl36a* | 0.0017957 | 0.772895451 | ENSDARG00000058105 |
| *rps15* | 0.00853213 | 0.763299742 | ENSDARG00000070849 |
| *rpl12* | 0.00165955 | 0.750548566 | ENSDARG00000006691 |
| *faua* | 0.00998911 | 0.750021747 | ENSDARG00000099022 |
| *eef1a1l1* | 0.00210108 | 0.740031897 | ENSDARG00000020850 |
| *eef1b2* | 0.00356875 | 0.702319451 | ENSDARG00000044521 |
| *txn* | 0.00374213 | 0.69743723 | ENSDARG00000031435 |
| *rpl19* | 0.00347454 | 0.682573297 | ENSDARG00000013307 |
| *vdrb* | 0.00386878 | 0.658963082 | ENSDARG00000070721 |
| *rps17* | 0.0086188 | 0.635588574 | ENSDARG00000104011 |
| *mgst1.2* | 0.00308202 | 0.618909833 | ENSDARG00000022165 |
| *slc20a1b* | 0.00390137 | 0.544320516 | ENSDARG00000010641 |
| *lxn* | 0.00040474 | 0.541893779 | ENSDARG00000043102 |
| *ubr5* | 0.00063043 | 0.540568381 | ENSDARG00000018192 |
| *gab1* | 0.00999364 | 0.502500341 | ENSDARG00000037018 |
| *fbxo11a.1* | 0.00022071 | 0.494764692 | ENSDARG00000102294 |
| *plekha7b* | 0.00063644 | 0.494764692 | ENSDARG00000062220 |
| *erlin1* | 0.00174488 | 0.494764692 | ENSDARG00000021991 |
| *meaf6* | 0.00470236 | 0.494764692 | ENSDARG00000101216 |
| *rasa1a* | 0.0064962 | 0.462971976 | ENSDARG00000035535 |
| *rnaseka* | 0.00120115 | 0.45169597 | ENSDARG00000069461 |

Supplementary Table 6: Top 30 differentiated marker genes of Gsubcluster1

| Gene | p_val | log2FC | Accession Number |
| --- | --- | --- | --- |
| *zgc:100868* | 4.82E-08 | 2.34103692 | ENSDARG00000004748 |
| *zgc:111983* | 3.55E-05 | 1.87266236 | ENSDARG00000006588 |
| *nrg1* | 0.00112902 | 1.79605325 | ENSDARG00000104314 |
| *icn2* | 0.00010115 | 1.71685802 | ENSDARG00000055514 |
| *spaca4l* | 3.50E-06 | 1.44057259 | ENSDARG00000116617 |
| *cyt1* | 0.0002359 | 1.43422548 | ENSDARG00000092947 |
| *si:dkey-19b23.12* | 6.55E-07 | 1.40839219 | ENSDARG00000100712 |
| *zgc:153665* | 0.00169878 | 1.35056756 | ENSDARG00000043442 |
| *hsd17b12a* | 0.00107697 | 1.27594189 | ENSDARG00000015709 |
| *epcam* | 9.01E-05 | 1.23399371 | ENSDARG00000040534 |
| *lmo7a* | 1.15E-05 | 1.22757887 | ENSDARG00000004930 |
| *krt4* | 0.00054844 | 1.18781516 | ENSDARG00000017624 |
| *cd9b* | 0.00024932 | 1.16046467 | ENSDARG00000016691 |
| *ptpn13* | 0.00056304 | 1.13661793 | ENSDARG00000103699 |
| *anxa2a* | 1.46E-05 | 1.12804319 | ENSDARG00000003216 |
| *flrt2* | 0.00570347 | 1.09487633 | ENSDARG00000079355 |
| *thrab* | 7.65E-06 | 1.07300183 | ENSDARG00000052654 |
| *si:ch211-125o16.4* | 0.00016753 | 1.06316747 | ENSDARG00000056836 |
| *icn* | 0.00273268 | 1.0614173 | ENSDARG00000009978 |
| *cldn23a* | 0.00032856 | 1.03708226 | ENSDARG00000028096 |
| *si:cabz01007794.1* | 0.00061393 | 1.03493379 | ENSDARG00000105590 |
| *evpla* | 0.00062619 | 1.02073991 | ENSDARG00000019808 |
| *ppp1r13l* | 1.02E-06 | 1.01236603 | ENSDARG00000013777 |
| *frmd4ba* | 4.21E-05 | 0.99053967 | ENSDARG00000074599 |
| *ahnak* | 0.00011629 | 0.98713307 | ENSDARG00000061764 |
| *si:ch211-157c3.4* | 0.00011459 | 0.97201958 | ENSDARG00000087093 |
| *nr1i2* | 0.00149024 | 0.95883081 | ENSDARG00000029766 |
| *tead3b* | 0.00268879 | 0.95764779 | ENSDARG00000063649 |
| *scel* | 0.0007324 | 0.95265979 | ENSDARG00000034677 |
| *znf185* | 6.01E-05 | 0.92230493 | ENSDARG00000103917 |

Supplementary Table 7: Top 30 differentiated marker genes of Gsubcluster2

| Gene | p_val | log2FC | Accession Number |
| --- | --- | --- | --- |
| *npffl* | 4.81E-07 | 4.83920379 | ENSDARG00000045016 |
| *aff2* | 7.35E-07 | 2.39346196 | ENSDARG00000052242 |
| *iglon5* | 2.83E-08 | 2.3348709 | ENSDARG00000022176 |
| *prl2* | 1.35E-05 | 2.28540222 | ENSDARG00000018744 |
| *chrm2a* | 4.04E-07 | 2.23026066 | ENSDARG00000098612 |
| *cntnap2a* | 2.56E-06 | 2.18586655 | ENSDARG00000058969 |
| *scg2b* | 8.34E-08 | 2.10852446 | ENSDARG00000038574 |
| *cacna1g* | 9.95E-06 | 2.10253816 | ENSDARG00000089913 |
| *nrxn3a* | 0.00120371 | 2.07389811 | ENSDARG00000043746 |
| *celf5a* | 1.29E-09 | 2.07199858 | ENSDARG00000071375 |
| *gpm6aa* | 5.82E-09 | 2.027906 | ENSDARG00000055455 |
| *elavl4* | 6.33E-06 | 1.97447312 | ENSDARG00000045639 |
| *ptprt* | 5.41E-05 | 1.83474081 | ENSDARG00000074781 |
| *grid1b* | 1.12E-06 | 1.77520749 | ENSDARG00000044161 |
| *rxfp1* | 2.45E-06 | 1.71836163 | ENSDARG00000090071 |
| *gabbr2* | 6.80E-07 | 1.6479723 | ENSDARG00000061042 |
| *unc5da* | 0.00012092 | 1.6479723 | ENSDARG00000092722 |
| *kcnma1a* | 5.05E-07 | 1.63332552 | ENSDARG00000079840 |
| *pbx3b* | 0.00459271 | 1.49839594 | ENSDARG00000013615 |
| *nfasca* | 0.00045183 | 1.48703608 | ENSDARG00000061099 |
| *cdh6* | 0.00025734 | 1.48243907 | ENSDARG00000014522 |
| *rtn1a* | 3.82E-05 | 1.44409996 | ENSDARG00000006497 |
| *gfra1a* | 1.70E-05 | 1.41970331 | ENSDARG00000099732 |
| *clstn1* | 5.62E-08 | 1.41664675 | ENSDARG00000031720 |
| *tmem269* | 3.55E-07 | 1.41372632 | ENSDARG00000079307 |
| *ctnna2* | 5.48E-06 | 1.38493789 | ENSDARG00000024785 |
| *msi2b* | 0.0001358 | 1.38324954 | ENSDARG00000032614 |
| *slc6a1b* | 0.00031252 | 1.357552 | ENSDARG00000039647 |
| *ptprn2* | 1.00E-08 | 1.35579155 | ENSDARG00000035970 |
| *celf2* | 4.88E-07 | 1.35579155 | ENSDARG00000002131 |

**Supplementary Figure 5**: UpSet plot illustrating the functional categorization of 22 genes in zebrafish GnRH3 neurons across five categories: GnRH neuron development (Dev), migration (Mig), regulation of GnRH secretion/function (Reg), axon guidance/synaptic connectivity (Guid), and Kallmann syndrome/congenital hypogonadotropic hypogonadism (KS/CHH). The plot presents both the total number of genes assigned to each category (left) and the sizes of their specific intersections (top), revealing predominant and unique patterns of functional overlap among gene subsets.


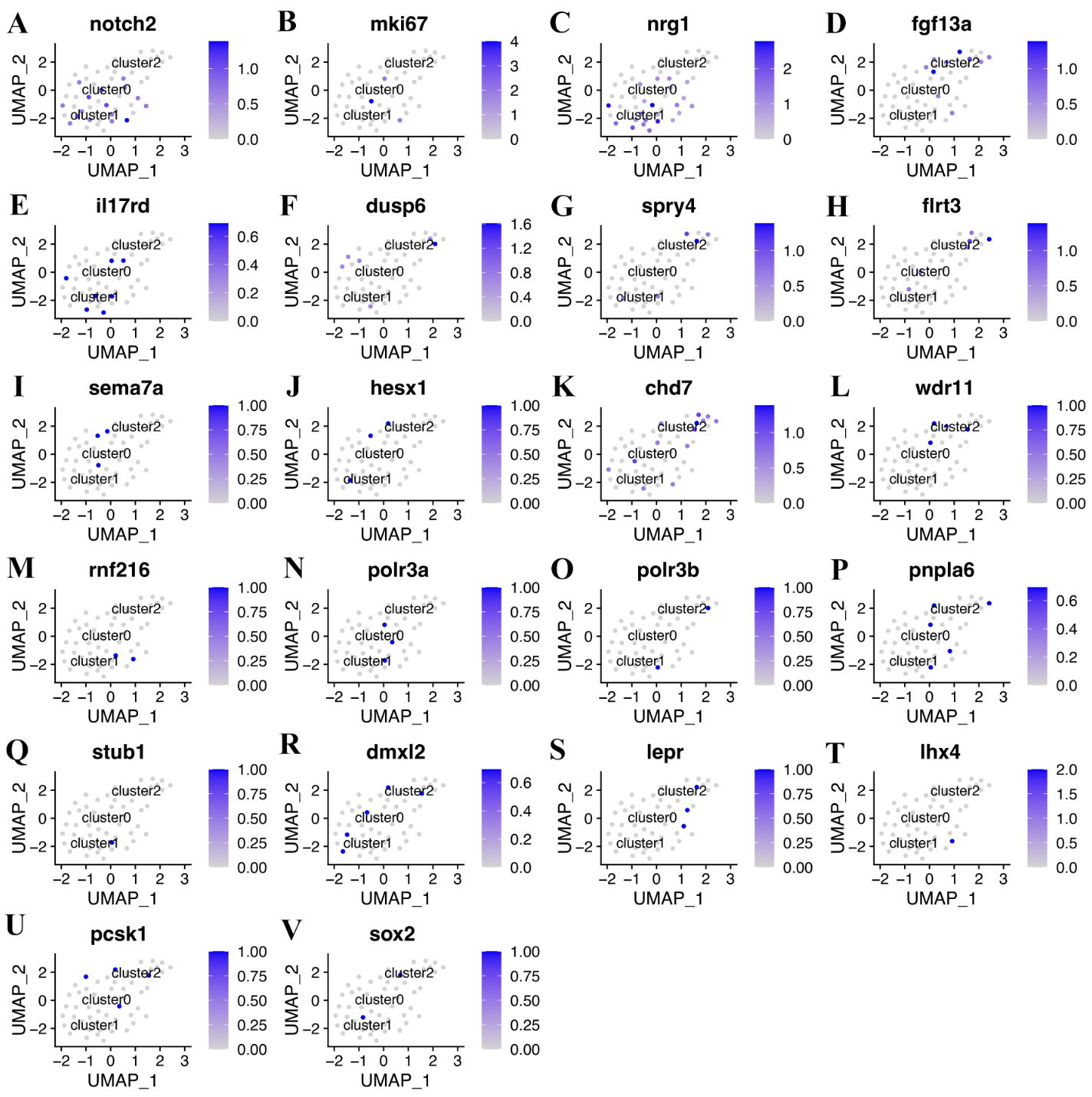


**Supplementary Figure 6**: Relative expression and distribution of specific genes involved in GnRH neuronal development and migration exhibit variable expression levels and distribution across different Gsubclusters.


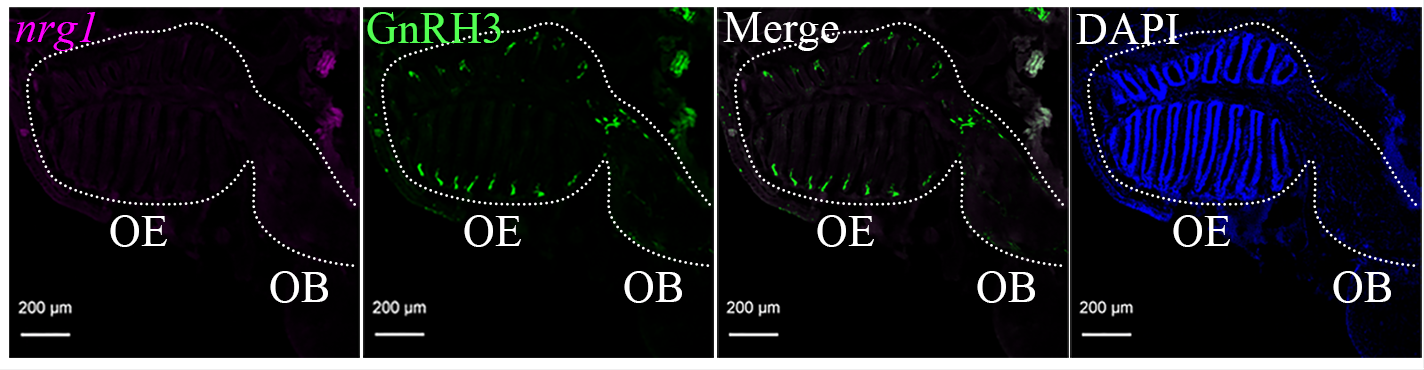


**Supplementary Figure 7**: Validation of NRG1 antibody specificity. Immunostaining was performed on mature male zebrafish in the absence of the primary NRG1 antibody to evaluate non-specific background signals. No detectable magenta fluorescence was observed under these conditions, confirming that the secondary antibody and imaging settings did not generate non-specific staining. These results verify the specificity of the NRG1 primary antibody used in Figure 7B. Green and blue channels showing GnRH3 and nuclear staining corresponding to the same field, respectively.

Supplementary Figure S8: FACS gating strategy and WT negative controls for enrichment of clean EGFP-positive nuclei from *Tg(gnrh3:EGFP)* zebrafish larvae. (A) FACS gating of 7 dpf WT nuclei stained with 7-AAD. WT nuclei lacking GFP expression were used to define the background GFP fluorescence and establish the GFP-positive gate. Only a minimal fraction of WT events fell within the DP/clean GFP-positive gate. (B) FACS gating of 3 dpf WT nuclei stained with 7-AAD, providing an additional GFP-negative control for background fluorescence. (C) FACS gating of 7 dpf *Tg(gnrh3:EGFP)* nuclei. Events were first gated based on FSC-A versus SSC-A to exclude debris, followed by FSC-A versus FSC-W gating to enrich singlets. Within the singlet population, 7-AAD-positive/GFP-positive events were selected using the double positive (DP) gate. A subsequent V450 versus GFP plot was used to distinguish auto-fluorescent events from clean GFP-positive nuclei. The auto-fluorescence gate was excluded, and the clean GFP-positive gate was used for sorting. The final clean GFP-positive gate was positioned away from the auto-fluorescence gate and used for downstream collection. FSC-A, forward scatter area; SSC-A, side scatter area; FSC-W, forward scatter width; V450-A, V450 fluorescence area.
